# Molecular phylogeny and dynamic evolution of disease resistance genes in the legume family

**DOI:** 10.1186/s12864-016-2736-9

**Published:** 2016-05-26

**Authors:** Fengya Zheng, Haiyang Wu, Rongzhi Zhang, Shiming Li, Weiming He, Fuk-Ling Wong, Genying Li, Shancen Zhao, Hon-Ming Lam

**Affiliations:** Centre for Soybean Research, Partner State Key Laboratory of Agrobiotechnology and School of Life Sciences, The Chinese University of Hong Kong, New Territories, Hong Kong; BGI-Shenzhen, Shenzhen, 518083 China; HKU-BGI Bioinformatics Laboratory and Department of Computer Science, University of Hong Kong, Pofulam, Hong Kong; Crop research institution, Shandong Academy of Agricultural Sciences, Jinan, 250100 China

**Keywords:** *R*-genes, Legumes, Evolution, **C**oiled-**c**oil (CC) domain, **T**oll/**I**nterleukin-1 **r**eceptor (TIR) domain, **N**ucleotide-**b**inding **s**ite (NBS), **L**eucine-**r**ich **r**epeat (LRR) domain

## Abstract

**Background:**

Legumes are the second-most important crop family in agriculture for its economic and nutritional values. Disease resistance (*R*-) genes play an important role in responding to pathogen infections in plants. To further increase the yield of legume crops, we need a comprehensive understanding of the evolution of *R*-genes in the legume family.

**Results:**

In this study, we developed a robust pipeline and identified a total of 4,217 *R*-genes in the genomes of seven sequenced legume species. A dramatic diversity of *R*-genes with structural variances indicated a rapid birth-and-death rate during the *R*-gene evolution in legumes. The number of *R*-genes transiently expanded and then quickly contracted after whole-genome duplications, which meant that *R*-genes were sensitive to subsequent diploidization. R proteins with the **C**oiled-**c**oil (CC) domain are more conserved than others in legumes. Meanwhile, other types of legume R proteins with only one or two typical domains were subjected to higher rates of loss during evolution. Although *R*-genes evolved quickly in legumes, they tended to undergo purifying selection instead of positive selection during evolution. In addition, domestication events in some legume species preferentially selected for the genes directly involved in the plant-pathogen interaction pathway while suppressing those *R*-genes with low occurrence rates.

**Conclusions:**

Our results provide insights into the dynamic evolution of *R*-genes in the legume family, which will be valuable for facilitating genetic improvements in the disease resistance of legume cultivars.

**Electronic supplementary material:**

The online version of this article (doi:10.1186/s12864-016-2736-9) contains supplementary material, which is available to authorized users.

## Background

The legume family, known as Fabaceae or Leguminosae, evolved about 60 million years ago (mya) [1–3]. The name of the legume family was derived from the multi-seeded structure of their fruits, which are known as legumes or pods [4]. At present, the legume family is the third-largest flowering plant family after Orchidaceae and Asteraceae. The species within the legume family are ecologically important because most of them are able to fix atmospheric nitrogen through symbiosis with nitrogen-fixing bacteria in their root nodules [5]. Legumes are also agriculturally important as they are used as major food crops, forage and green manure. For example, soybean, peanut and chickpea together account for more than 20 % of the primary crop production worldwide [6]. As plant diseases could cause a great loss of crop production, researches on disease resistance are becoming more and more important in the legume family.

Recent advances in DNA sequencing technologies have resulted in tremendous progress in both plant and animal studies [7]. Recently, lots of efforts have been made in the whole-genome sequencing of legumes. The genome sequences of some species in the legume family are now publicly available, including cultivated soybean (*Glycine max*) [8], wild soybean (*Glycine soja*) [9, 10], barrel clover (*Medicago truncatula*) [11], bird’s-foot trefoil (*Lotus japonicus*) [12], pigeonpea (*Cajanus cajan*) [13], chickpea (*Cicer arietinum*) [14], and common bean (*Phaseolus vulgaris*) [15]. A total of 950 megabases (Mb) of genome sequences of cultivated soybean were assembled with 46,430 high-confidence protein-coding genes. The soybean genome experienced an early legume-specific whole-genome duplication (WGD) ~59 mya and a soybean-specific WGD ~13 mya [8]. An assembly of 868-Mb genome sequences of wild soybean was also published, which represents 74.2 % of the estimated 1.17-Gb genome [9]. For barrel clover, 375-Mb high quality genome sequences with 44,124 gene models were anchored onto eight pseudo-molecules by optical mapping and fluorescence in-situ hybridization [11]. The 315-Mb genome of bird’s-foot trefoil contained 34,245 protein-coding genes. Of them, about 10,951 genes have complete structures, whereas 19,848 are partial genes. The 605-Mb pigeonpea and 544-Mb chickpea genomes were also available at present, with 48,680 and 28,269 predicted genes, respectively [13, 14]. The common bean genome project achieved the assembly of a 472.5-Mb sequence with 27,197 protein-coding genes [15]. These sequenced genomes provide us with data resources for genome-wide analyses of *R*-genes in the legume family.

It is well known that all long-lived organisms need an immune system that is characterized by high specificity, self-tolerance and immune memory [16]. Plants have evolved different but sophisticated immune strategies from animals to protect themselves from various pathogen attacks. Numerous *R*-genes reported in different plants have typical domains and motifs, which are ancient and highly conserved in gymnosperms, flowering plants and animals [17, 18]. A large number of plant R proteins contain two characteristics: a **n**ucleotide-**b**inding **s**ite (NBS) and a C-terminal **l**eucine-**r**ich **r**epeat (LRR) region. The NBS is part of a central NB-ARC (**n**ucleotide-**b**inding adaptor shared by **A**PAF-1 [apoptotic protease-activating factor 1], **R** proteins, and **C**ED-4 [the *Caenorhabditis elegans* homolog]) domain [19]. The central NB-ARC domain consists of three subdomains, which are the nucleotide-binding subdomain and two ARC subdomains. LRR proteins play a central role in the growth and developmental processes of plants, such as hormone perception, organ formation, and immune response [20]. LRR domains were predicted to interact directly with their effectors and determine recognition specificity. Modifications of the LRR structure may disturb R protein-effector interactions and alter effector recognition specificities [20, 21]. Plant NBS-LRR-encoding genes with different N-termini act as protein–protein interaction cassettes and are involved in downstream signaling responses. The N-terminal domain can be divided into two main subclasses, which are the **T**oll/**I**nterleukin-1 **r**eceptor homology region (TIR) domain and the **C**oiled-**c**oil (CC) domain. Based on the N-terminal extensions, the NBS-LRR proteins can then be categorized into the TIR-NBS-LRR (TNL) subclass and the CC-NBS-LRR (CNL) subclass [16, 17, 22].

The structures of *R*-genes are highly diverse according to comparative genomic analyses in vertebrates and plants. Evolutionary studies suggested *R*-gene families as some of the most plastic families in plants, which were associated with intense structural shuffling leading to synteny erosion [23]. What is more, tandem and segmental duplications are thought to contribute to the structural plasticity of NBS-LRR domains in different plant genomes [24, 25]. Intensive genomic studies on *R*-genes have been reported in Arabidopsis, grasses and other angiosperm species [17, 26, 27]. Recently, NBS-coding *R*-genes were investigated in four legume species based on either BAC or genome sequences [28–31]. However, until now, how the different types of *R*-genes evolved across the legume family, especially under natural and artificial selections, has remained elusive. Thus, the objectives of this research are: 1) to identify *R*-genes in currently sequenced legume species; 2) to elucidate the structures of *R*-genes in legumes; 3) to infer the birth and death rates of *R*-genes during the dynamic evolution of legumes; and 4) to detect the selection signals in the *R*-genes during the evolution and domestication of some legumes.

## Results and discussion

### Phylogenetic analyses of sequenced legume species

In order to understand the characteristics of disease resistance genes of the sequenced species within the legume family, we downloaded the genome sequences and gene models of the seven legume species, including cultivated soybean, wild soybean, barrel clover, bird’s-foot trefoil, pigeonpea, chickpea, and common bean from the public databases (Additional file 1: Table S1 and Table S2). We also downloaded the genome and gene sequences of grape, which was used as the out-group species in our analyses, as grape represents the basal rosid lineage and has close-to-ancestral karyotypes that facilitate comparisons across major eurosids [32, 33]. Genome sizes of these seven legumes vary from 315 Mb to 1.1 Gb, and the numbers of genes range from 28,269 to 46,430, as a result of different evolutionary processes and genome qualities [34].

**Fig. 1:**
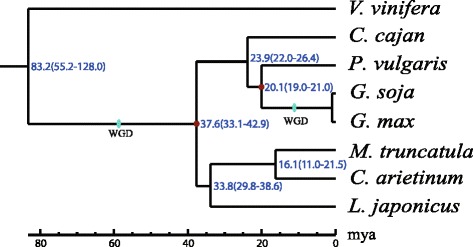
The phylogenetic tree of the legume species with grape as the out-group. The red dot represents the calibration time point. WGD: whole-genome duplication; mya: million years ago; Cultivated soybean: *Glycine max*; Wild soybean: *Glycine soja*; Barrel clover: *Medicago truncatula*; Bird’s-foot trefoil: *Lotus japonicus*; Pigeonpea: *Cajanus cajan*; Chickpea: *Cicer arietinum*; Common bean: *Phaseolus vulgaris;* Grape: *Vitus vinifera*

The legume family, Fabaceae, is divided into these subfamilies: Caesalpinioideae, Mimosoideae, and Papilionoideae/Faboideae. One of the better known members within the Papilionoideae subfamily is the genus *Glycine*. It consists of two subgenera: *Soja* and *Glycine*. Subgenus *Soja* consists of two annual self-pollinated species: the cultivated soybean, *Glycine max*, and its wild progenitor, *G. soja*, while the other subgenus *Glycine* comprises more than a dozen wild perennial species [35]. Most cultivated legumes are categorized within the millettioid/phaseoloid clade and the hologalegina clade (galegoids, cool-season legumes). Here, the phylogenetic tree of legumes was constructed with genome-wide single-copy orthologous genes by concatenating four-fold degenerate sites of each single-copy family to one supergene (Fig. 1).

The divergence of grape and Papilionoids was estimated to be 83.2 mya, and the legume species diverged from one another about 37.6 mya, which was earlier than a previous estimation [30]. Based on the fossil records, the divergence of Fabales from the closest group Rosales and Cucurbitales was inferred to be at 59.9 mya. The divergence of Rosids and Asterids was 89.3 mya and then a Papilionoideae-specific WGD was observed among these legumes. In addition, soybean underwent an additional recent soybean lineage-specific palaeotetraploidization at ~13 mya [8]. Barrel clover, pigeonpea and bird’s-foot trefoil separated from one another ~33.8 mya. Soybean, pigeonpea and common bean evolved from a common ancestor ~23.9 mya.

The distribution of gene clusters among the seven sequenced legumes and grape showed that there were more multiple-copy orthologs than single-copy orthologs (Additional file 2: Figure S1A). It suggested that additional WGD’s could have contributed to the increased number of orthologous genes. More unique paralogs were observed in barrel clover than in other legumes. Similarly, more unique paralogs were identified in the wild than the cultivated soybean. We also assessed the gene families shared among wild and cultivated soybean, chickpea and barrel clover (Additional file 2: Figure S1B). A total of 9,531 gene families were shared among these four species, with 606, 932, 572, and 2,525 gene families being specific to cultivated soybean, wild soybean, chickpea and barrel clover, respectively.

### Identification of *R*-genes in legumes

Disease resistance genes are an important component of the plant immune system [36, 37]. We developed a robust pipeline to identify *R*-genes and their homologs in the sequenced legumes (Additional file 3: Figure S2). With the pipeline, a total of 4,217 *R*-genes were identified in the seven legumes: 227 from chickpea, 815 from pigeonpea, 744 from cultivated soybean, 952 from wild soybean, 270 from bird’s-foot trefoil, 770 from barrel clover, and 439 from common bean (Additional file 4: Dataset S1). Generally, more *R*-genes were identified here than previously reported, which could be attributed to the improved pipeline with iterative searches and more types of R proteins as seeds from the Plant Resistance Gene database (PRGdb) [38].

No apparent positive correlation could be found between the genome size and the number of *R*-genes. The number of *R*-genes annotated from the original gene models was compared with those predicted from our self-curated database (Additional file 5: Figure S3). The higher proportion of annotated *R*-genes from the original gene models in common bean, barrel clover, and cultivated soybean were probably a result of the high-quality gene prediction in the genome sequencing projects. However, fragmentary genome assemblies could make some *R*-genes break up into more partial ones, leading to higher *R*-gene numbers.

A total of 952 and 744 *R*-genes were identified in wild and cultivated soybeans, respectively (Additional file 1: Table S2). Compared to its cultivated counterpart, wild soybean has an additional 208 *R*-genes, indicating that many *R*-genes could have been lost during the soybean domestication process. It also suggested that *R*-genes underwent rapid gain-and-loss events during evolution. However, the number of *R*-genes might be over-estimated in wild soybean due to the fragmentary assembly [9].

R proteins were classified into four distinct groups based on their domains: NBS, LRR, TIR, and CC (Table 1). The R proteins with N-terminal CC motifs or TIR motifs before NBS domains, and C-terminal LRR domains, were categorized using Pfamscan, Marcoil and InterProScan [39–41]. The corresponding *R*-gene sequences were semi-manually checked for typical domains and the R proteins were categorized according to the conserved features. As a result, 76.1 %, 33.8 %, 24.2 %, and 29.5 % of the R proteins were identified in legumes containing NBS, LRR, CC and TIR domains, respectively. As a comparison, 65.1 %, 45.5 %, 23.8 %, and 15.1 % of the R proteins identified in grape contain NBS, LRR, CC and TIR domains, respectively. Except for those categories listed here, *R*-genes are frequently found as chimeric genes with other additional domains (Additional file 4: Dataset S1), which triggered a new decoy model for the activation of these chimeric *R*-genes [42, 43].

**Table 1:** Categories of *R*-genes according to the typical domains in the legume family and grape

	Cultivated soybean	Wild soybean	Barrel clover	Bird’s-foot trefoil	Pigeonpea	Chickpea	Common bean	Grape	Total
CC	19	13	12	2	111	6	11	7	181
CC-NBS	46	62	44	26	41	31	40	75	365
CC-NBS-LRR	68	47	49	11	37	19	31	69	331
CC-TIR-NBS	6	6	20	5	5	3	1	-	46
LRR	26	83	44	10	30	21	34	76	324
NBS	156	213	193	82	136	51	59	182	1072
NBS-LRR	70	58	102	18	56	15	20	130	469
TIR	53	112	44	19	39	17	57	75	416
TIR-NBS	62	76	127	53	36	8	8	7	377
TIR-NBS-LRR	67	49	44	16	47	6	1	14	244
Others	104	108	56	18	152	19	62	90	608
Un-annotated	67	126	35	10	125	31	35	29	458
Total	744	952	770	270	815	227	359	754	4891

Note: Cultivated soybean: *Glycine max*; Wild soybean: *Glycine soja*; Barrel clover: *Medicago truncatula*; Bird’s-foot trefoil: *Lotus japonicus*; Pigeonpea: *Cajanus cajan*; Chickpea: *Cicer arietinum*; Common bean: *Phaseolus vulgaris*; Grape: *Vitus vinifera*

Nepal et al. reported 188 CNL type *R*-genes, which is higher than our results, in cultivated soybean [31]. The authors only used MEME algorithm [44] to identify CC domains, while we employed other softwares and databases as mentioned above to limit the false positive predictions. We characterized 475 NBS-coding *R*-genes in cultivated soybean and 579 in barrel clover, which are quite close to the reported 465 and 571 for the two species [30]. Similar approaches and parameters were used in both studies, and yet iterative searches could identify kinds of *R*-genes as more as possible. These researches contributed more NBS type *R*-genes than the 319 ones identified by Kang et al. [29], which could probably be benefit from updated soybean annotations and more R proteins in PRGdb.

Interestingly, the number of R proteins with the TIR domain experienced a distinct expansion in dicots when compared to monocots [45]. In our analysis, R proteins with the TIR domain make up a higher proportion in legumes than in grape, indicating that the number of R proteins with TIR domains might have experienced additional expansions after the divergence of legumes from other dicots. The lineage-specific expansion of TIR-domain R proteins might have diversified the resistance functions in combination with R proteins containing other domains in the legume species.

### The landscape of *R*-genes in legumes

**Fig. 2:**
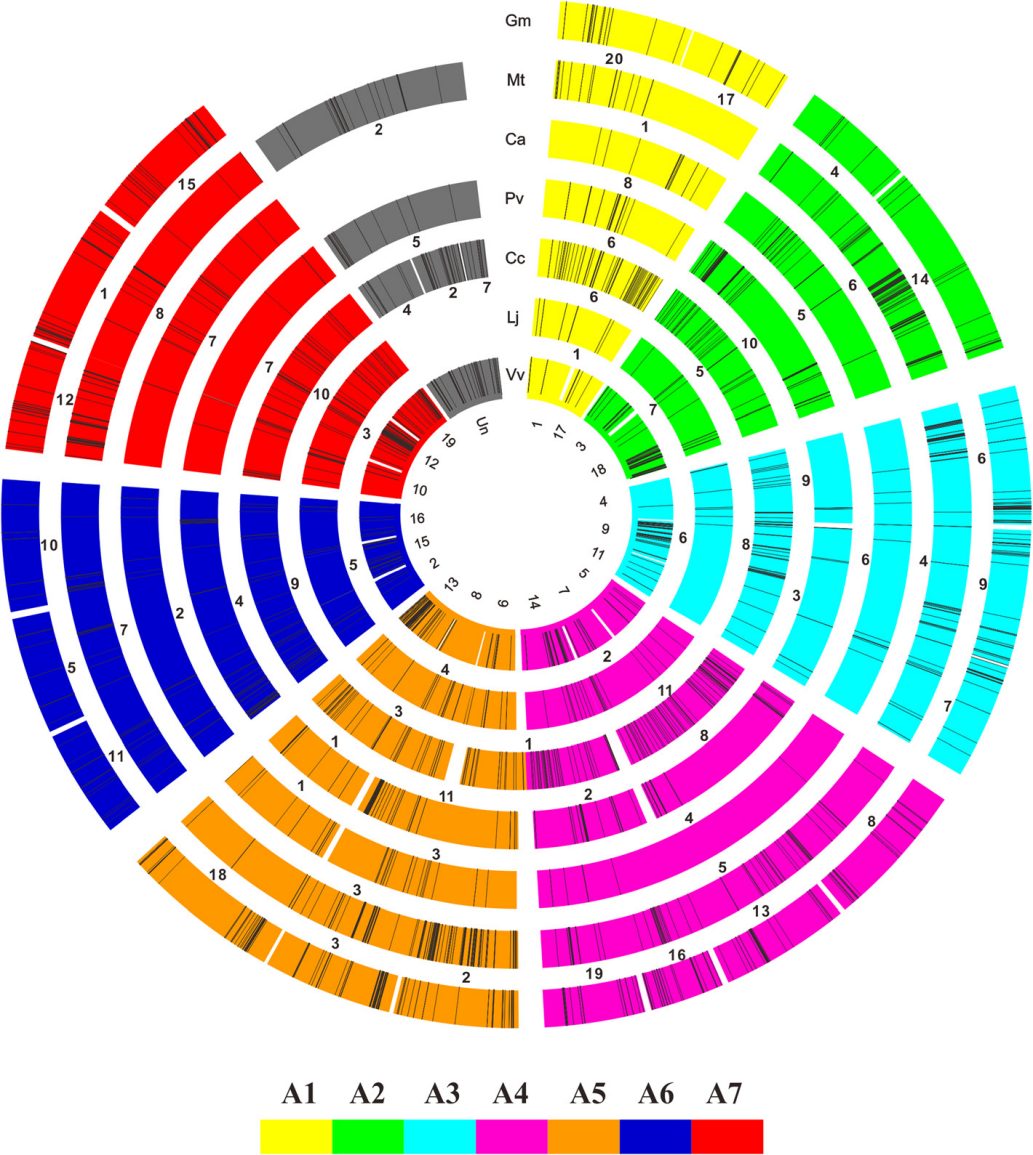
Integrative *R*-gene landscape in legumes using grape as ancestral species. Genome synteny is illustrated as concentric circles. The chromosomes are highlighted with a color code that represents the legume ancestral genome structure (A1 to A7, inner circle). The loci of *R*-genes on different chromosomes are marked as black bars. Gm: *Glycine max*; Mt: *Medicago truncatula*; Ca: *Cicer arietinum*; Pv: *Phaseolus vulgaris*; Cc: *Cajanus cajan*; Lj: *Lotus japonicus*; Vv: *Vitus vinifera*

The average length of R proteins in legumes is ~500 amino acids (Additional file 6: Figure S4), with the average lengths in wild soybean and barrel clover being much higher than the rest. To obtain an integrated *R*-gene map, the *R*-genes from different species were aligned to the grape chromosomes based on synteny analyses (Fig. 2). Protein sequences from the seven legume species were mapped to the grape protein sequences, and then orthologous genes were determined between each legume species and grape. Using orthologous genes, the chromosomes of each legume species was anchored to the corresponding chromosomes of grape. The ancestral relationship was then represented with the visualization tool Circos [46]. Based on the synteny, an integrated *R*-locus map was generated for the legume family. The visualized figure showed a remarkable syntenic presence or absence of polymorphism of *R*-genes in the legume family (Fig. 2). Compared with protein-coding genes, *R*-genes show a reduced syntenic conservation during the legume evolution.

*R*-genes are distributed unevenly and tend to localize on different chromosomes in different legume species (Fig. 2; Additional file 7: Figure S5). This might have been caused by duplication events or chromosome rearrangements [47]. For example, among the 20 chromosomes of cultivated soybean, Chr18 carries most of the *R*-genes, whereas Chr04 has the fewest (85 *vs.* 11). Chr10 of common bean contains most of the *R*-genes, whereas Chr09 carries the fewest (115 *vs.* 2). No obvious pattern was discovered in the distribution of different *R*-gene categories by structure. We constructed a phylogenetic tree of all the *R*-genes identified in legumes (Additional file 8: Figure S6). Four major groups were found in the tree, reflecting the evolutionary dynamics and cross-species relationships of *R*-genes in legumes. Of them, Group I and Group II are far from each other, whereas the other two are much closer in the phylogenetic tree.

### Structural variances of *R*-genes

The evolution of nucleotides and amino acids has been studied a lot using sequence alignments, but much less attention has been paid to the evolution of gene structures [48]. Previous studies indicated that gene structures changed over time, just like what happened in amino acid sequences [49]. The identification of *R*-genes in the legume species here provides a panoramic perspective for investigating the evolution of *R*-gene structures on a variety of timescales starting from the origin of the legume family. Therefore, we constructed a pipeline using GeneWise to refine and rectify *R*-gene structures [50].

Due to complex evolutionary causes and potential errors in genome assembly, a significant portion of *R*-genes are only partial or have frame-shift/nonsense point mutations (Table 2). Setting aside those *R*-genes with intact structures, we classified the remaining *R*-genes into pseudo-genes on the basis of frame-shift variations, as well as putative functional genes, including those lacking a start codon, or lacking a stop codon, or lacking both. *R*-genes would be regarded as pseudo-genes if an open reading frame shift happened in the coding sequences. Manual annotation detected that 7-30 % of *R*-genes experienced pseudogenization in the legume family. Our results support the conclusion that changes in the intron–exon structure are gradual, clock-like, and largely independent of coding-sequence evolution [51, 52].

**Table 2:** Summary of the different *R*-gene structures in the legume family and grape

Species	Complete	InDel	Lack start codon	Lack stop codon	Lack start and stop codons	Pseudo
Cultivated soybean	202	169	97	91	212	142
Barrel clover	454	48	90	80	89	57
Bird’s-foot trefoil	40	85	31	27	106	66
Pigeonpea	234	184	138	21	330	92
Chickpea	96	29	26	8	62	35
Common bean	17	86	105	14	117	106
Grape	246	133	140	47	164	157

Note: Cultivated soybean: *Glycine max*; Barrel clover: *Medicago truncatula*; Bird’s-foot trefoil: *Lotus japonicus*; Pigeonpea: *Cajanus cajan*; Chickpea: *Cicer arietinum*; Common bean: *Phaseolus vulgaris*; Grape: *Vitus vinifera*

The structure variations of *R*-genes are always chimeric in plants, as what we discovered in our analysis [53]. *R*-genes tend to gather in a cluster due to tandem duplications, recombination hotspots, or active transposon elements [45]. Using OrthoMCL [54], we identified a total of 372 *R*-gene families in legumes, which varied from 48 in bird’s-foot trefoil to 200 in wild soybean (Additional file 1: Table S3). Of these, 302 *R*-gene families are specific to legumes. If two *R*-genes are no more than eight genes apart, they were defined as a cluster in our analyses. About 12-76 % of the *R*-genes on average exist as clusters in legumes (Additional file 1: Table S4). In barrel clover, 76 % of the *R*-genes are clustered, which is much higher than the figures in the other legumes (Additional file 9: Figure S7). Compared to other legumes, a lower percentage of *R*-genes with singleton domains was observed in barrel clover and bird’s-foot trefoil. This difference could also reflect the structure variance among the legumes, which might be associated with the genome sizes, or large-scale genome structural variations. Possible, harsh survival environments stimulated the expansion of *R*-genes in legumes by tandem duplication to increase the dosage effects.

### The birth and death rates of *R*-genes in legume evolution

**Fig. 3:**
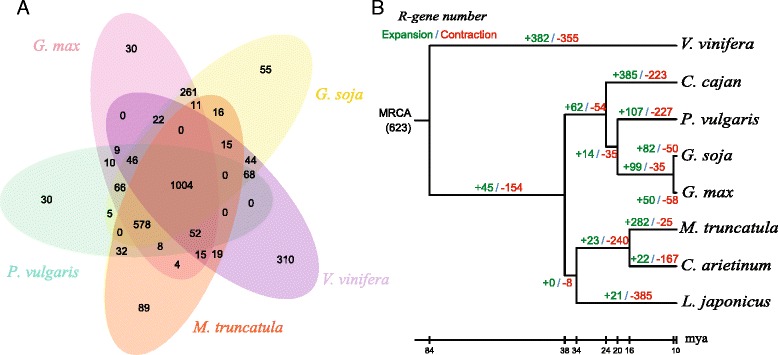
The births and deaths of *R*-genes during the legume evolution. **a** A Venn diagram showing common *R*-genes among grape (*V. vinifera*), barrel clover (*M. truncatula*), wild soybean (*G. soja*), cultivated soybean (*G. max*) and common bean (*P. vulgaris*); **b** The birth and death rates of *R*-genes during the process of evolution. The red and green colors denote the expansion and contraction of *R*-gene numbers at each divergence event in the phylogenetic tree, respectively. MRCA, most recent common ancestor. mya: million years ago

Disease resistance genes have rapid birth and death rates in plants as they evolved and interacted with pathogens [31, 55]. Remarkable differences in *R*-gene numbers have been shown among legumes. In this study, we analyzed the conserved and species-specific *R*-genes among cultivated and wild soybeans, common bean, barrel clover and grape (Fig. 3a). Most of the *R*-gene families are conserved in legumes but diverged from grape. A total of 1,004 *R*-genes are conserved among these five species, and 578 are lineage-specific in the four legumes. Significantly more specific genes were observed in grape than in the four legume species, which suggests a recent gene radiation from a common ancestor of the legumes.

To further understand the expansion and contraction of *R*-genes, we constructed the phylogenetic tree with birth and death events at different stages of the legume evolution. There were 623 and 514 *R*-genes in the common ancestor of dicots and legumes, respectively (Fig. 3b; Additional file 1: Table S5). The birth and death of *R*-genes has remained stable in grape, which coincides with the fact that no WGD event happened in grape. About 60 mya, the legume branch went through a WGD event, during which *R*-genes also experienced a rapid expansion. However, *R*-genes suffered a large-scale contraction in legumes during the following 20 million years. This contraction may have followed the diploidization event after the WGD in legumes. Since then, the number of *R*-genes had decreased to 514 in the common ancestor of legumes ~38 mya. After the divergence of legume species, *R*-genes experienced a dramatic expansion and contraction with a high birth-and-death rate. For example, a lot of *R*-genes were lost in bird’s-foot trefoil, chickpea and common bean, whereas an obvious expansion of *R*-genes happened in pigeonpea and barrel clover. Our investigation also revealed that some *R*-genes originated after the divergence of legumes. The frequent births and deaths of *R*-genes in legumes suggested their highly distinct evolutionary pattern.

However, a subsequent WGD did not result in a large-scale increase in *R*-genes in wild and cultivated soybeans. Instead, many *R*-genes were lost in the diploidization process, which is similar to the scenario happened in the tetraploid legume ancestor. Our results support the hypothesis that *R*-genes were sensitive to diploidization after WGD events. However, a net increase of 32 *R*-genes was detected in wild soybean, while eight more *R*-genes were lost in cultivated soybean. Thus, the birth and death of *R*-genes in soybeans might be mediated by artificial selection during domestication.

Significantly, most of the eudicots have experienced one or up to three ancient WGD events [56]. WGD events are a major driving force for the evolution of protein-coding genes, especially for the dosage-dominant genes such as transcription factors and microRNA genes [57]. Here, we provided evidence for *R*-genes also being dosage-sensitive or diploidization-sensitive in legumes. An examination of the expansion and contraction of *R*-genes also showed that their numbers did not significantly increase after the legume-specific WGD and the soybean-specific WGD. On the contrary, tandem duplications played an important role in the increase of these *R*-genes. The dosage effect can be compensated for by a reshuffling recovery mediated by tandem duplication, transposition, and recombination, etc. Thus, *R*-genes were flexible enough to adapt to diverse environments in a very short time.

### Distinct evolution of typical R protein domains

**Fig. 4:**
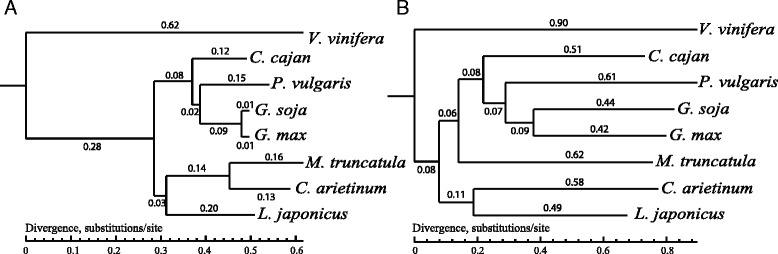
Divergence rates of genome-wide (**a**) single-copy genes and (**b**) *R*-genes. The branch length as denoted by the number represents the lineage-specific neutral substitution rate

To compare the evolutionary rate of *R*-genes with other protein-coding genes, phylogenetic trees with substitution rates were constructed in our analyses (Fig. 4). For each species, we concatenated four-fold degenerate sites of single-copy genes into one supergene and tested different substitution models. The polygenetic tree based on *R*-genes was built with *R*-gene families that cover all the eight species (seven legume species plus grape). The topology of species tree inferred by protein-coding genes (Fig. 4a) is very similar to that of the *R*-gene tree (Fig. 4b). However, the average divergence rate of *R*-genes was twice as high as that of genome-wide genes. The branch lengths of the *R*-gene tree are much longer than those of the genome-wide single-copy gene tree with many more substitutions per site, which supports our hypothesis that *R*-genes evolved much faster than the average genome-wide genes. This is to be expected for the disease resistance system to be able to adapt quickly to variable environments to increase the fitness of plants.

To further investigate the evolutionary rates of different *R*-genes, we constructed the divergence trees of different R proteins with TIR-NBS, CC-NBS, CNL (CC-NBS-LRR), TNL (TIR-NBS-LRR) and NBS-LRR domains (Additional file 10: Figure S8). The average divergence rates of R proteins with the CNL domain are much lower than those with the CC-NBS and NBS-LRR domains (Additional file 11: Figure S9). The R proteins with single NBS domains evolved much faster than other kinds of R proteins. R proteins with the CNL domain have lower evolutionary rate as the carboxy-terminal domains of CNLs are smaller and less varied than those of TNLs. In wild soybean and bird’s-foot trefoil, R proteins with NBS-LRR domains evolved faster than those with other domains, while those with CC-NBS domains had the fastest substitution rates in chickpea.

R proteins with the typical CNL and TNL structures have CC and TIR motifs in the N-terminus, respectively. R proteins with the TNL structure are common in dicots but are absent or at least rare in monocots [58]. In legumes, R proteins with the CNL domain evolved more slowly than those with the TNL domain, indicating that *R*-genes encoding proteins with the CC domain tended to be retained during evolution. On the other hand, the average divergence rates of NBS-LRR-encoding genes were highly variable, being much higher in wild soybean, bird’s-foot trefoil and pigeonpea than those in cultivated soybean, common bean, chickpea and barrel clover. It showed that *R*-genes encoding the NBS-LRR domain evolved quickly, which contributed to the rapid overall birth-and-death events of *R*-genes.

To defend against infections by pathogens, plants have adopted *R*-genes for intracellular surveillance, which encode proteins that can recognize various pathogen effectors and initiate rapid effector-triggered immunity. For instance, an *R*-gene encoding an R protein with the TNL structure was demonstrated to confer symbiotic specificity, and this gene was later identified as a PHASE locus mediated by miR482 in soybean and barrel clover [59]. This indicates that the interactions between miRNA and *R*-genes might have long-term evolutionary benefits by buffering NBS-LRR levels to reduce the fitness cost of these genes. The co-evolution of miRNAs and *R*-genes may also have resulted in the lower evolutionary rate of CNL and TNL domains.

### Selection signals of *R*-genes in legume evolution and domestication

**Fig. 5:**
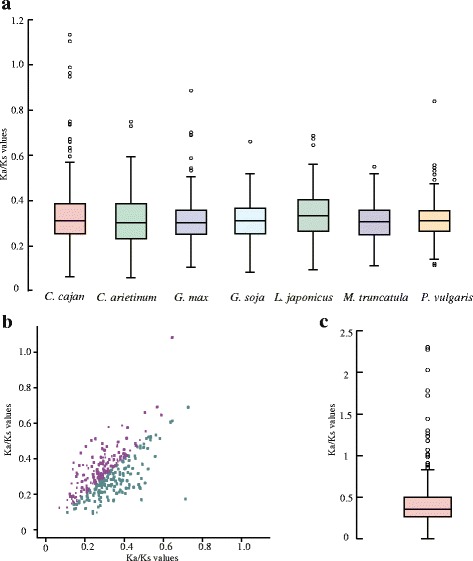
The selection signals of *R*-genes in legumes. **a** The boxplot of Ka/Ks values of pairs of *R*-genes between each legume species and grape; **b** The scatter plot of Ka/Ks values of pairs of *R*-genes between wild soybean (purple), cultivated soybean (green) and barrel clover; **c** The boxplot of Ka/Ks values of pairs of cultivated and wild soybean *R*-genes

A fundamental measure of the relative importance of selection in causing amino-acid substitution is the ratio of non-synonymous to synonymous substitutions. To detect selection signals in *R*-genes, we calculated the Ka/Ks ratio of *R*-gene pairs between each legume and the out-group, grape (Fig. 5a). When some non-synonymous mutations are deleterious and the rest neutral, the Ka/Ks ratio will be less than one. A value of Ka/Ks < 0.25 thus indicates the signal of purifying selection. Our results showed that 32 % of the *R*-genes in legumes seemed to have undergone purifying selection. Typically, a value of Ka/Ks > 1 indicates the operation of positive selection in causing some amino-acid substitutions. We only detected two candidates with Ka/Ks > 1 and five others with Ka/Ks between 0.8 and 1.0 in our analysis. These *R*-genes probably underwent positive selection, or some substitutions of them could also be caused by genetic drift.

As barrel clover is an important model within legumes, we further used barrel clover as the out-group to detect selection signals in soybean (Fig. 5b). It could provide some clues of adaptation during soybean evolution. Only one *R*-gene showed strong positive selection in wild soybean. Weak instead of strong signals of positive selection were observed in cultivated soybean. These results provided clues that, although *R*-genes evolved quickly in legumes, they seemed to have avoided positive selection during evolution. Most *R*-genes were subjected to purifying selection, which probably constrained *R*-gene evolution.

Modern soybean cultivars were originally domesticated from its wild progenitor, an endemic species in China, more than 3000 years ago [60]. The cultivated and wild soybeans exhibit very different adaptation strategies such as different resistance against different pathogens. The Ka/Ks ratios of gene pairs were calculated between cultivated and wild soybeans (Fig. 5c). We found that 21 *R*-gene pairs with Ka/Ks > 0.8 were potentially affected by artificial or natural selection (Additional file 1: Table S6). The purifying selection in cultivated soybeans was also stronger than that in wild soybeans. However, population-scale re-sequencing analysis could be utilized to check whether the positive selection signals were caused by adaptation in wild soybeans or by domestication in cultivated soybeans.

Wild soybean lives in complex natural environments with such challenges as pests, drought and salt stress [61–63]. In cultivated soybean, the strong pressure from artificial selection impelled the fixation of favorable traits in a founder population within a short turnaround time. Re-analyzed the results from previous study [64], we know that very few *R*-genes were affected by artificial selection during domestication. Among them, only two orthologs of the *RPS2* (*R*esistance to *Pseudomonas syringae 2*) gene encoding the CNL domain showed strong signals of artificial selection. Instead, 37 genes involved in plant-pathogen pathways were strongly selected by artificial selection. Thus, most of the *R*-genes with large Ka/Ks values were probably caused by adaptation or by genetic drift in wild soybeans.

Artificial selection during domestication may constrain the *R*-gene evolutionary rate. Novel *R*-genes occurring with low frequencies could be rapidly removed from the breeding population by the strong pressure of artificial selection. On the other hand, in wild soybean, the low frequency of genes introduced by random genetic drift could easily be fixed in the genome, once the plants have obtained the ability to successfully defend against pathogens. Besides, the genetic effects of *R*-genes are usually subtle in the defense responses to infection of plant pathogens, which may be invisible for artificial selection. As a result, genes directly involved in the plant-pathogen interaction pathway, rather than the general *R*-genes, tended to be strongly favored by soybean domestication.

## Conclusions

In our analyses, we provided a comprehensive understanding of the evolution of *R*-genes in sequenced legumes. In legumes, *R*-genes experienced a rapid birth-and-death rate with transient expansions and contractions during whole-genome duplications, indicating *R*-genes were sensitive to subsequent diploidization. Different domains of *R*-genes had distinct evolutionary rates, while the CNL-domain R proteins are more conserved in legumes. *R*-genes tended to undergo purifying selection instead of positive selection during evolution. Artificial selection appeared to have favored genes directly involved in the plant-pathogen interaction pathway, rather than typical *R*-genes, during domestication.

## Methods

### Identification of *R*-genes in legumes

The latest genome sequences and gene models of *Glycine max* (cultivated soybean), *Medicago truncatula* (barrel clover), *Lotus japonicus* (bird’s-foot trefoil), *Cajanus cajan* (pigeonpea), *Glycine soja* (wild soybean), *Cicer arietinum* (chickpea), *Phaseolus vulgaris* (common bean), and *Vitis vinifera* (grape) were downloaded from the public databases, NCBI and Phytozome. The versions of genome assembly and annotation used for each legume species were provided in Additional file 1: Table S2. To identify *R*-genes in these seven legume species, we modified a universal pipeline based on the HMM model and BLAST searches (Additional file 3: Figure S2). First, we retrieved the protein sequences of each species based on the genome annotations. The protein sequences were then mapped and trained against the model of the NB-ARC domains of Pfam profile (PF00931) using hmmer3.0 with default parameters.

To contain as many known *R*-genes as possible in the validating database, we downloaded sequences from the plant resistance gene database, PRGdb (www.prgdb.org) [38]. Those proteins with NB-ARC domains were further validated using a self-curated *R*-gene database by searching GeneBank with the key words, “ATP binding cassette”, “NBS”, “NBS-LRR”, “disease resistance genes”, and “LRR kinase”. The sequences of the genes encoding those species-specific proteins with typical features of *R*-genes were treated as seed sequences and aligned as queries to the corresponding genome using tblastn [65].

All significant hits (E-value <1e-10) from each species were mapped to the validating database. Only those proteins with the best hits were retained and considered as seed sequences. The *R*-genes were manually curated and verified if they had significant hits with any R protein in the constructed protein database. We carried out several iterations using the same approach described above, until no additional R proteins could be identified in each species. The species-specific proteins and those with the best hits were all considered as putative R proteins in the subsequent analyses.

### Analyses of typical *R*-gene domains

To characterize the putative *R*-genes, we comprehensively integrated the protein function prediction tools such as PfamScan [39], InterProscan [41, 66], and MARCOIL [40]. The NB-ARC, LRR, and TIR domains were identified using PfamScan with Pfam profile (PF00931, PF00560, PF01582) and InterProscan against corresponding InterPro10 entries. We used the MARCOIL program to identify CC motifs with a threshold probability of 50.

### Construction of a syntenic *R*-gene map

Protein sequences derived from the grape genome were used as subjects, and those from the seven legume species were mapped to the subjects as queries using BLASTP [65]. The orthologous genes between each legume species and grape were identified using the cumulative identity percentage (CIP) metrics. Only those genes with mapping CIP > 60 % were defined as orthologous gene pairs. The closest orthologous genes in the legume family can be identified by BLAST with the best hits of E-values <1e-10. If two genes were reciprocal best hits in a BLAST search, they were considered as alleles/orthologs [54]. We then calculated the orthologous genes on each chromosome of each legume species versus the grape coordinates. Based on these orthologous pairs, we could anchor the chromosomes of each legume species to the grape chromosomes. Then, we marked the location of *R*-genes on each chromosome with the software Circos [46]. Most of the *R*-gene loci identified in legumes can be mapped while those *R*-genes with synteny values lower than 3 could not be mapped. In this map, two or more *R*-genes that were separated by no more than eight genes were treated as a cluster.

### Sequence alignments and phylogenetic analyses

The phylogenetic tree of the legume species was constructed with genome-wide single-copy orthologous genes. For each species, we concatenated four-fold degenerate sites of each single-copy gene family to one supergene sequence. To obtain the single-copy genes, gene families were defined according to the putative protein sequences of each legume species using OrthoMCL [54]. Multiple alignments of amino acid sequences were performed using ClustalW with default options [67], and then phylogenetic trees were constructed based on the neighbor-joining method with a Kimura 2-parameter model by MEGA (version 6.0) [68]. The stability of internal nodes was determined by bootstrap analyses with 1,000 replicates.

### Analyses of *R*-gene structures

The sequences of *R*-genes were aligned to the corresponding reference genome using tblastn [65] with the threshold E value of 1e-5. The outputs in M8 format were extracted and only the best hits were selected for structural analyses. Based on the best hits, *R*-gene structures were re-annotated using the GeneWise software [50]. The different structures of the *R*-genes were identified, such as open-reading frame shift, premature stop codon mutations, or insertion/deletions. Additional file 3: Figure S2 illustrates the detailed pipeline we designed for the structural analyses of *R*-genes in the legume family.

### Birth and death rate of *R*-genes

Computational Analysis of gene Family Evolution (CAFE) [69] was used to detect the birth and death rate of *R*-genes in the legume family. The result of OrthoMCL [54] for all *R*-genes was reformatted and used as the input in CAFE (version 2.1). The key parameters are “*P*-value threshold 0.05, number of random 10,000, and search for the λ value”. *R*-gene clusters with *P* values <0.05 were analyzed manually. Then we summarized the contraction and expansion at each node of the divergence tree, which reflects the birth and death rate of *R-*genes.

### Detection of selection signals

To detect positive selections in each legume, the single-copy gene families were further used to calculate the Ka/Ks values. The codon sequences were obtained by aligning those sequences with MUSCLE [70]. The non-synonymous and synonymous nucleotide substitutions were calculated based on the Nei–Gojobori method with Jukes–Cantor correction [71]. The nucleotide divergence among orthologous genes was estimated by D_xy_ with the Jukes and Cantor correction in MEGA (version 6.0) [68, 72].

Grape was used as the out-group and the Ka/Ks value of each gene pair was calculated with a module in PAML named YN00 [73, 74]. After that, values from genetic models were also used as the quality control to check whether the model was appropriate. Only the values that were available and stable in different models were chosen in our analyses. Boxplots were used to show the Ka/Ks values.

## Additional files

Additional file 1: Table S1. Overview information of the seven published genomes in the legume family. **Table S2.** The genome assembly and number of identified *R*-genes in the seven legume species and grape. **Table S3.** The number of *R*-genes in each *R*-gene family in seven species of legumes and grape. Each row represents one *R*-gene family. **Table S4.** Global statistics of *R*-genes in families or clusters in seven species of legumes and grape. **Table S5.** Statistics on the expansions and contractions of *R*-genes during the evolution of legumes. **Table S6.** The gene pairs with outlier Ka/Ks values between wild and cultivated soybeans. The threshold for an outlier Ka/Ks value was set at 0.8. (XLSX 29 kb) Additional file 2: Figure S1. The orthologous gene families within the legumes with grape as the out-group. (A) Category of gene orthologs in legumes; (B) A Venn diagram showing the number of genes common among wild soybean (*G. soja*), cultivated soybean (*G. max*), barrel clover (*M. truncatula*) and chickpea (*C. arietinum*). (PDF 428 kb) Additional file 3: Figure S2. A schematic workflow of the pipeline for *R*-gene identification. (PDF 366 kb) Additional file 4: Dataset S1. Details and sequences of *R*-gene identified in each species. Grape (Vv): *Vitis vinifera*; Cultivated soybean (Gm): *Glycine max*; Wild soybean (Gs): *Glycine soja*; Barrel clover (Mt): *Medicago truncatula*; Bird’s-foot trefoil (Lj): *Lotus japonicas*; Pigeonpea (Cc): *Cajanus cajan*; Chickpea (Ca): *Cicer arietinum*; Common bean (Pv): *Phaseolus vulgaris*. (XLSX 1716 kb) Additional file 5: Figure S3. The number of annotated and newly predicted *R*-genes in each legume species. Annotated: the *R*-genes annotated from original gene models; Predicted: the *R*-genes predicted based on the R proteins from our self-curated database. (PDF 249 kb) Additional file 6: Figure S4. Boxplot showing the lengths of R proteins identified in legumes. The length of an R protein was expressed in number of amino acid residues (aa). (PDF 141 kb) Additional file 7: Figure S5. Chromosomal distributions of *R*-genes in the legume family. The different colors represent different species, and the Y-axis denotes the number of *R*-genes on each chromosome. Note that the legumes have different chromosomes and some genome assembly was not anchored to chromosomes. (PDF 591 kb) Additional file 8: Figure S6. The phylogenetic tree of all *R*-genes identified in legumes. Different colors represent *R*-genes with different typical domains. (PDF 2674 kb) Additional file 9: Figure S7. Percentage of *R*-genes (a) in clusters and (b) with singleton domains including NBS, LRR, TIR and CC. (PDF 436 kb) Additional file 10: Figure S8. The phylogenetic tree of *R*-genes with typical domains showing the different divergence rates among legumes. The numbers above the lines indicate the divergence rates of different *R*-genes with (A) NBS-LRR, (B) TIR-NBS, (C) CC-NBS-LRR, and (D) CC-NBS domains. (PDF 480 kb) Additional file 11: Figure S9. The average neutral divergence rates of different domains in *R*-genes in the legume family. Note that some domains have identical values that there are some overlapping points in the figure. (PDF 93 kb)

## Abbreviations

CC: Coiled-coil
CIP: Cumulative Identity Percentage
HMM: Hidden Markov Models
LRR: Leucine-Rich Repeat
LTR: Long Terminal Repeat
mya: million years ago
NBS: Nucleotide-Binding Site
TIR: Toll/Interleukin-1 Receptor
WGD: Whole Genome Duplication
