# Supplementary figures and images for: Molecular phylogeny and dynamic evolution of disease resistance genes in the legume family
Source: BMC Genomics. 2016 May 26;17:402. doi: 10.1186/s12864-016-2736-9 (PMC4881053; doi:10.1186/s12864-016-2736-9)

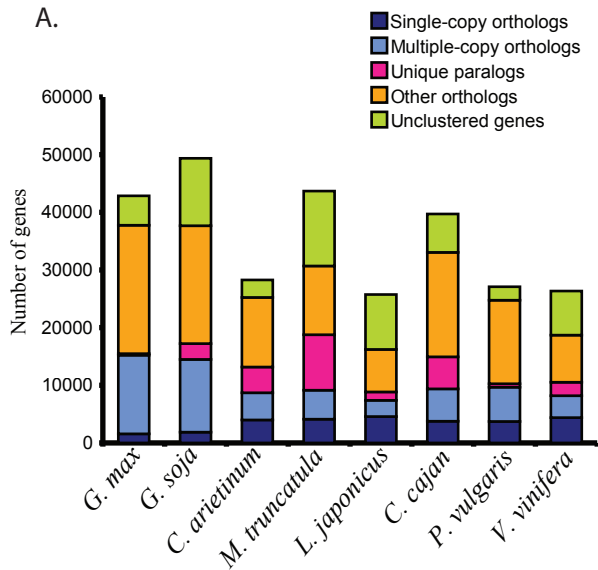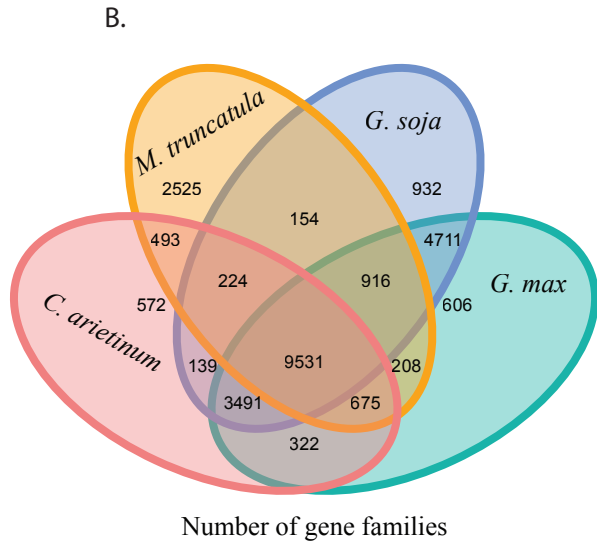

Supplement: Additional file 2: Figure S1. — The orthologous gene families within the legumes with grape as the out-group. (A) Category of gene orthologs in legumes; (B) A Venn diagram showing the number of genes common among wild soybean (G. soja), cultivated soybean (G. max), barrel clover (M. truncatula) and chickpea (C. arietinum). (PDF 428 kb) [file 12864_2016_2736_MOESM2_ESM.pdf]

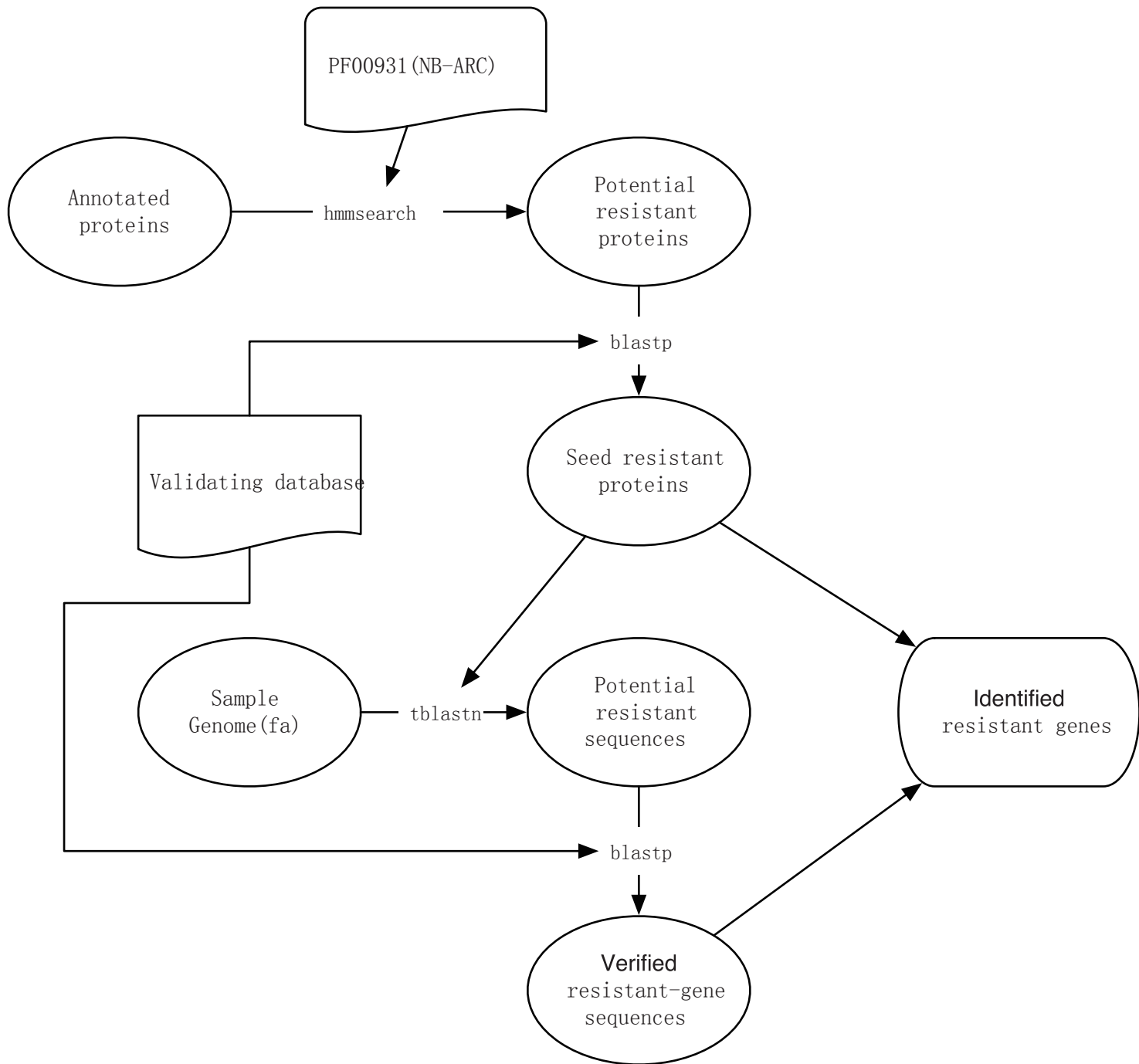

Supplement: Additional file 3: Figure S2. — A schematic workflow of the pipeline for R-gene identification. (PDF 366 kb) [file 12864_2016_2736_MOESM3_ESM.pdf]

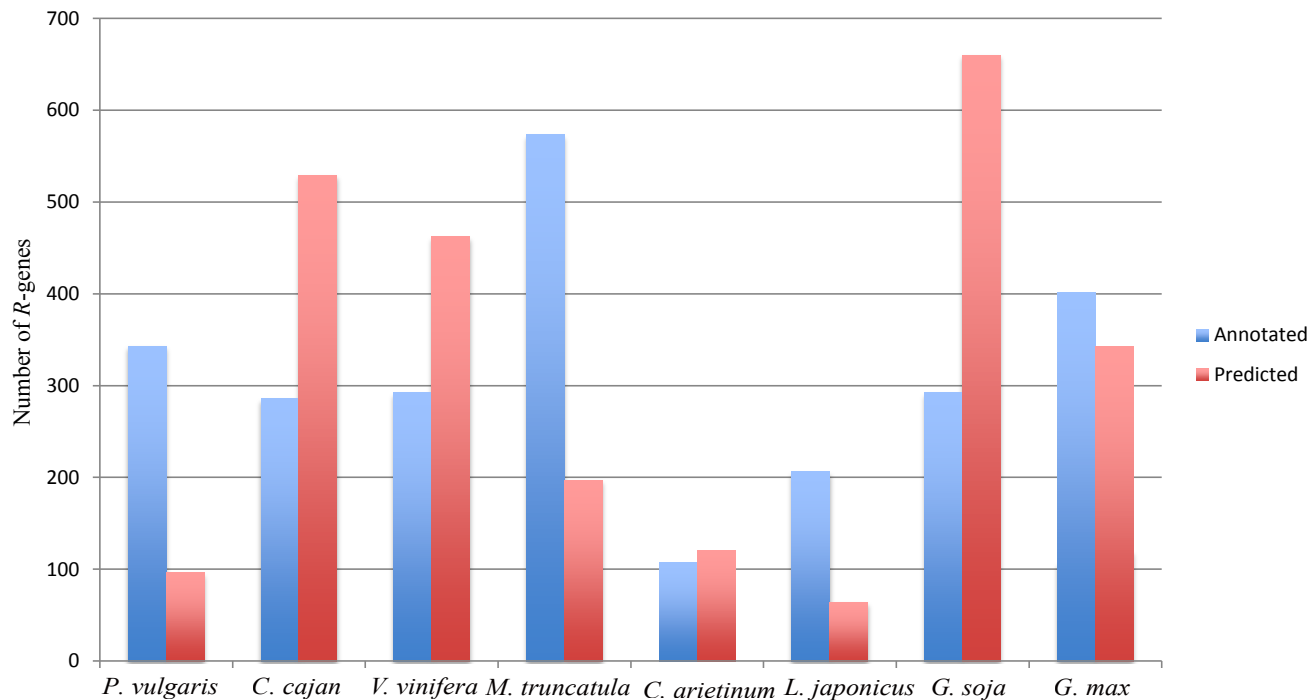

Supplement: Additional file 5: Figure S3. — The number of annotated and newly predicted R-genes in each legume species. Annotated: the R-genes annotated from original gene models; Predicted: the R-genes predicted based on the R proteins from our self-curated database. (PDF 249 kb) [file 12864_2016_2736_MOESM5_ESM.pdf]

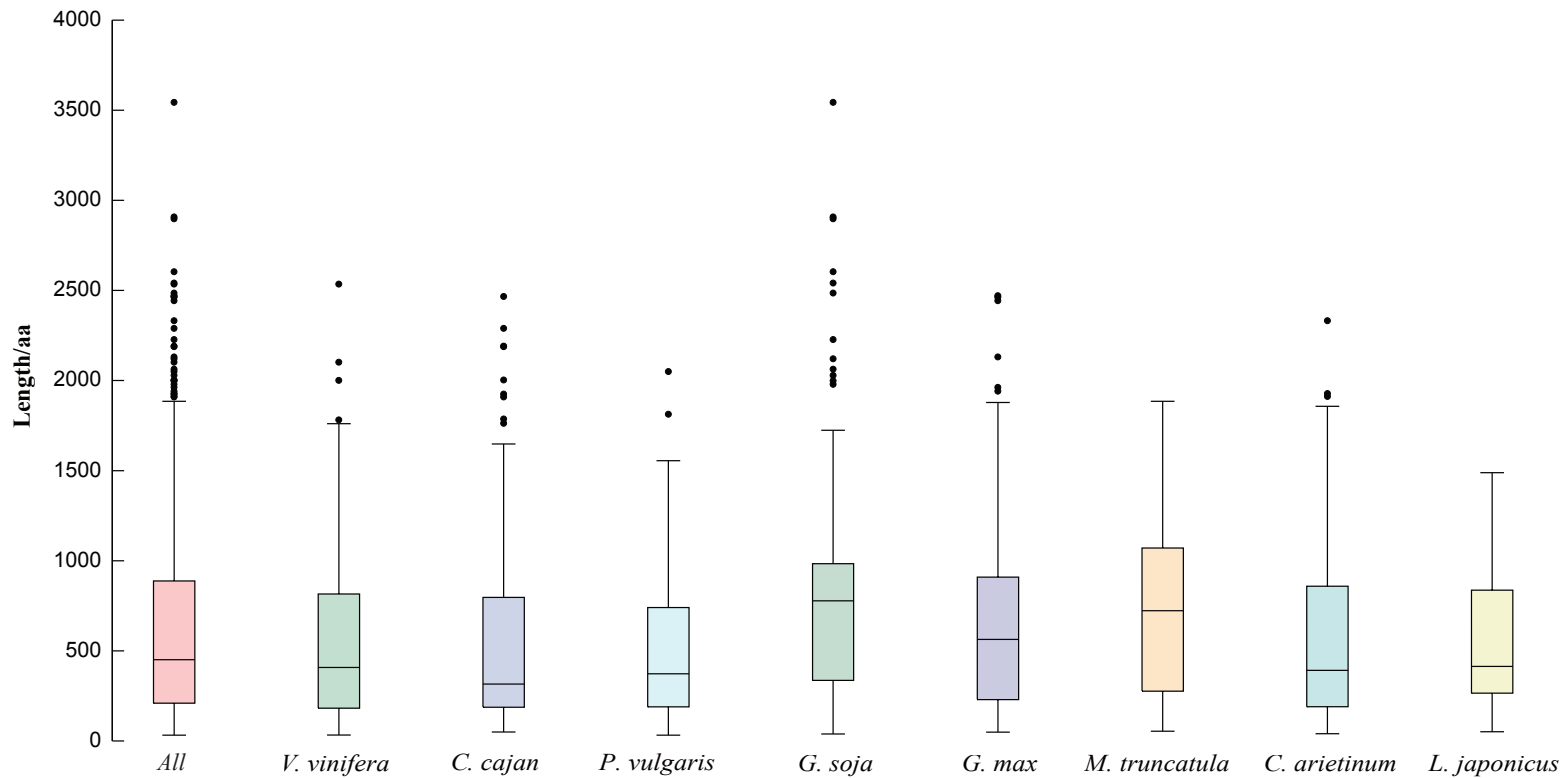

Supplement: Additional file 6: Figure S4. — Boxplot showing the lengths of R proteins identified in legumes. The length of an R protein was expressed in number of amino acid residues (aa). (PDF 141 kb) [file 12864_2016_2736_MOESM6_ESM.pdf]

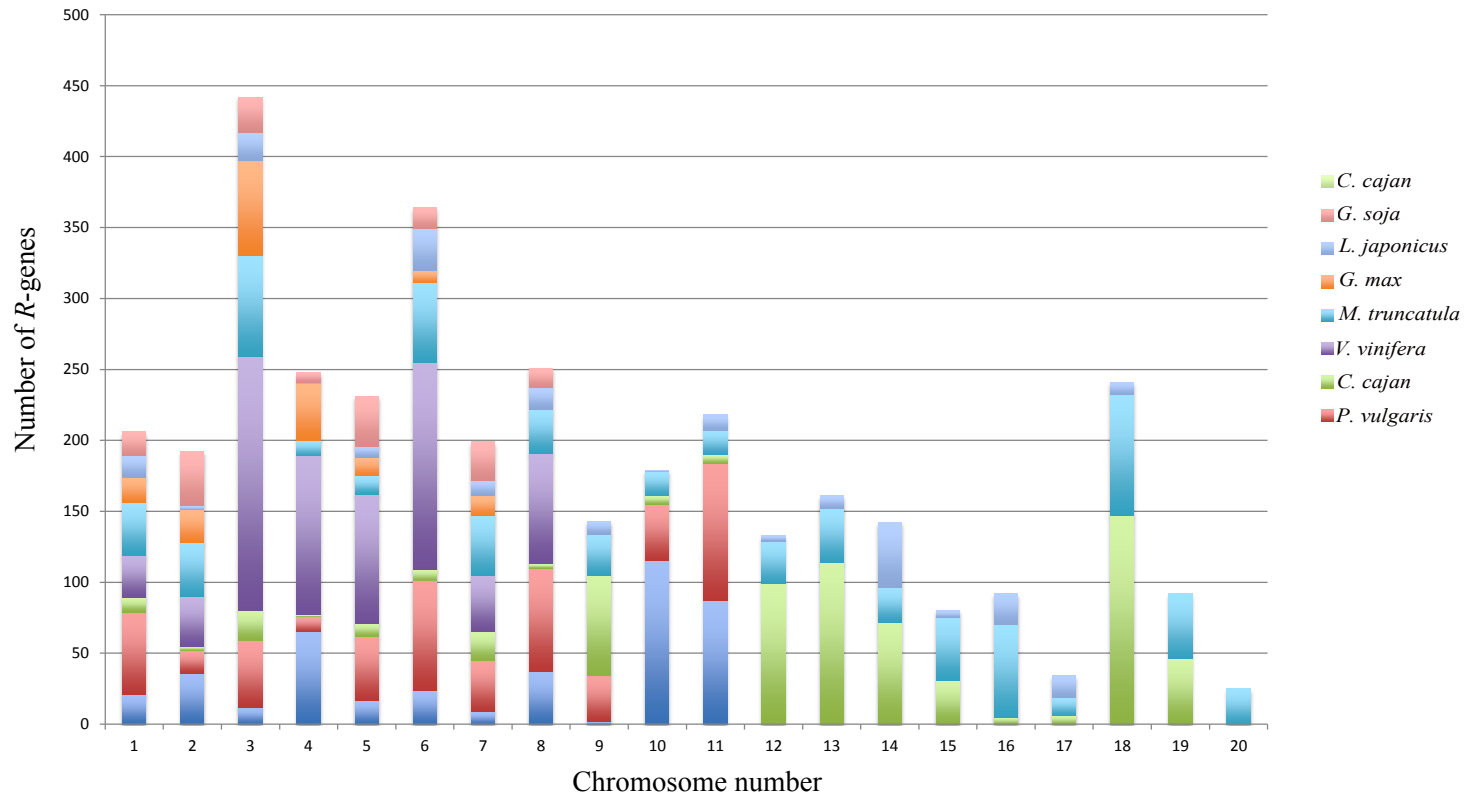

Supplement: Additional file 7: Figure S5. — Chromosomal distributions of R-genes in the legume family. The different colors represent different species, and the Y-axis denotes the number of R-genes on each chromosome. Note that the legumes have different chromosomes and some genome assembly was not anchored to chromosomes. (PDF 591 kb) [file 12864_2016_2736_MOESM7_ESM.pdf]

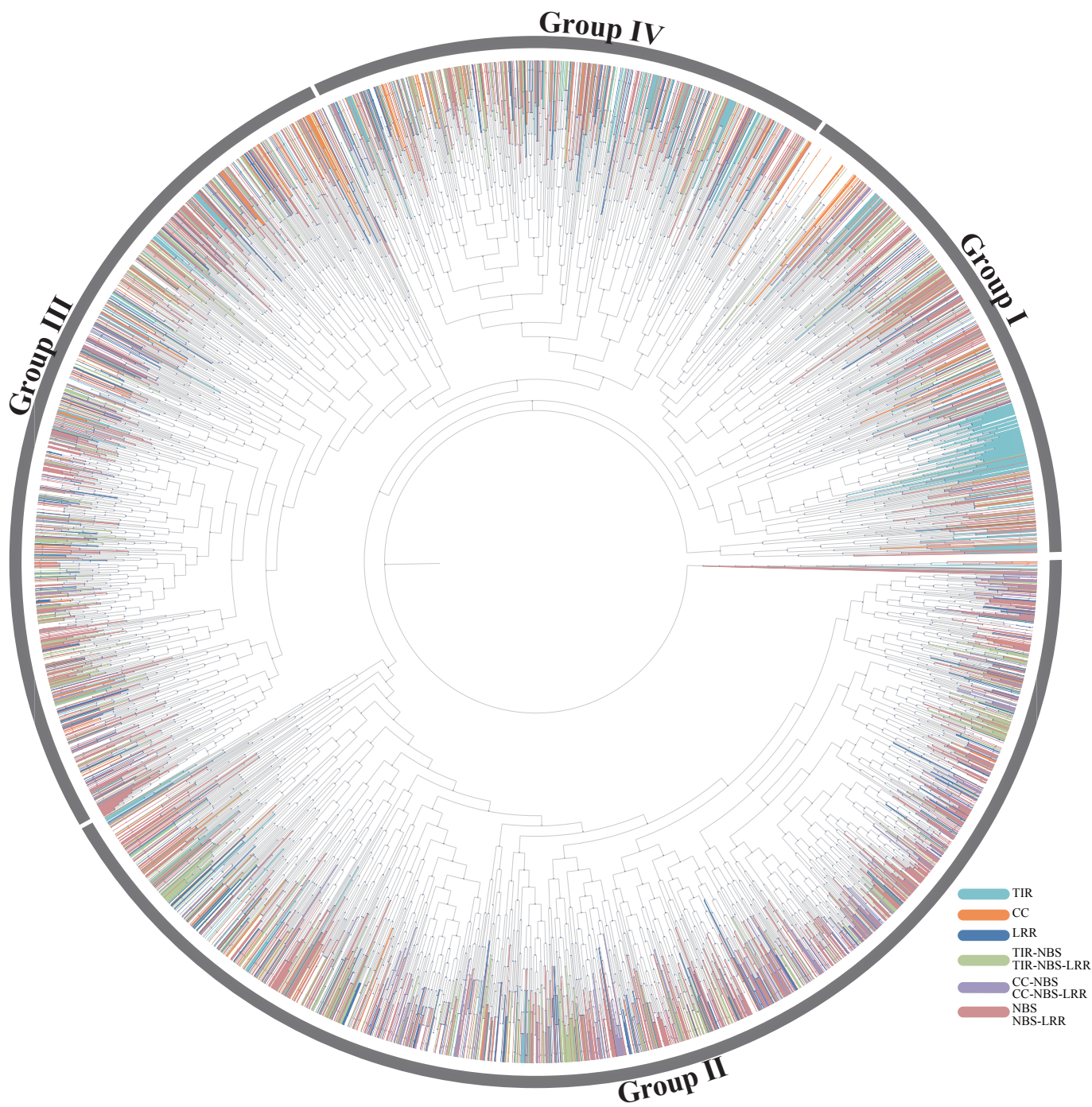

Supplement: Additional file 8: Figure S6. — The phylogenetic tree of all R-genes identified in legumes. Different colors represent R-genes with different typical domains. (PDF 2674 kb) [file 12864_2016_2736_MOESM8_ESM.pdf]

a.

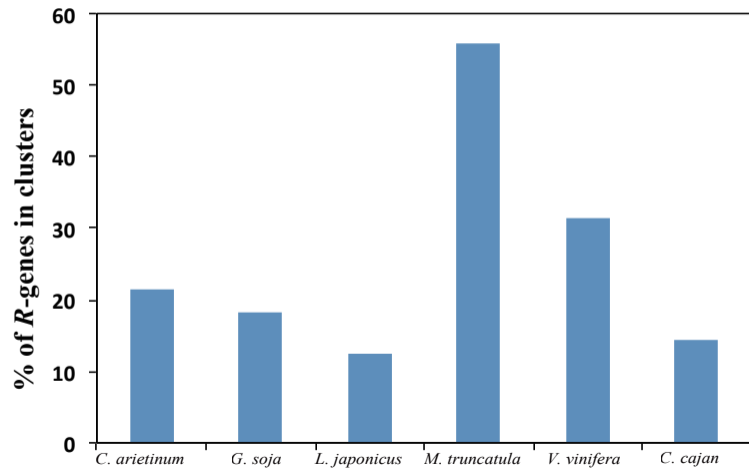

b.

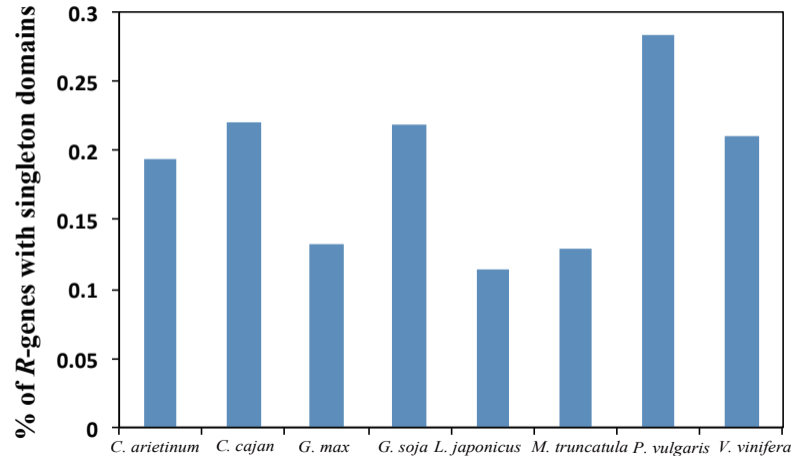

Supplement: Additional file 9: Figure S7. — Percentage of R-genes (a) in clusters and (b) with singleton domains including NBS, LRR, TIR and CC. (PDF 436 kb) [file 12864_2016_2736_MOESM9_ESM.pdf]

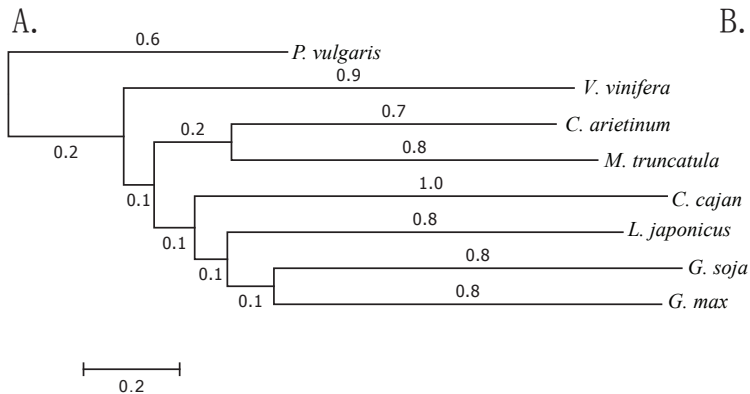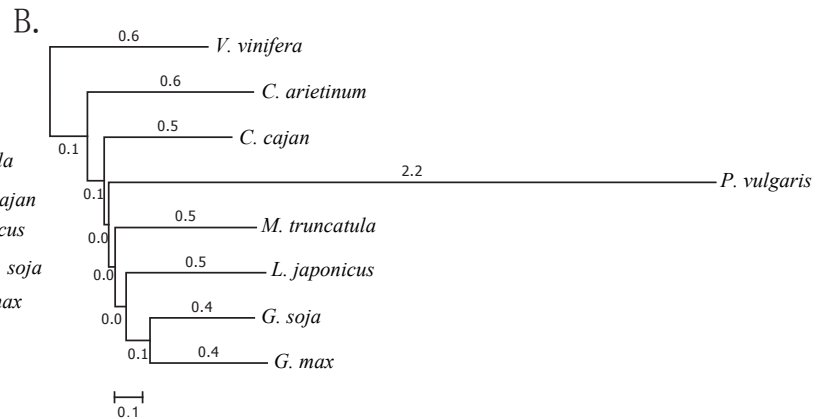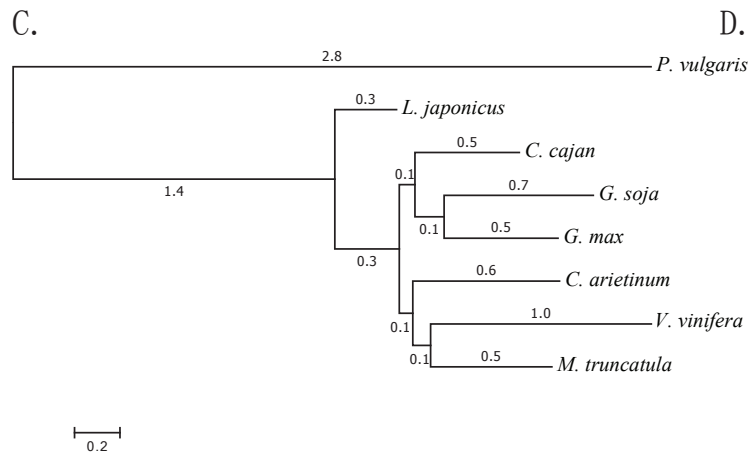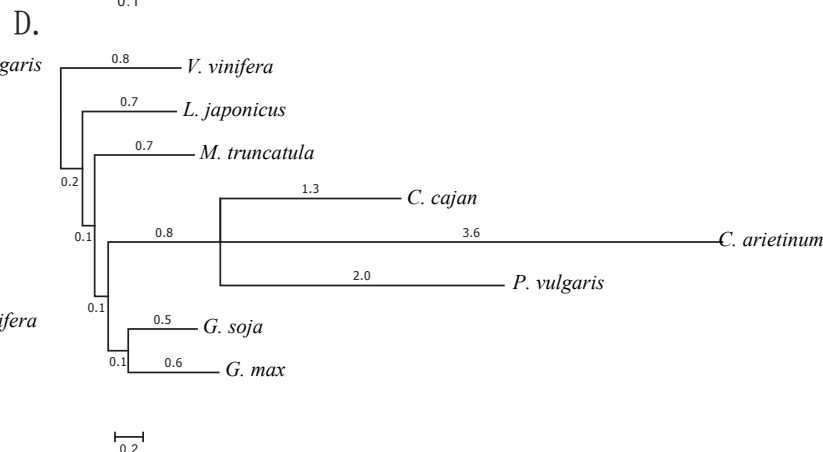

Supplement: Additional file 10: Figure S8. — The phylogenetic tree of R-genes with typical domains showing the different divergence rates among legumes. The numbers above the lines indicate the divergence rates of different R-genes with (A) NBS-LRR, (B) TIR-NBS, (C) CC-NBS-LRR, and (D) CC-NBS domains. (PDF 480 kb) [file 12864_2016_2736_MOESM10_ESM.pdf]

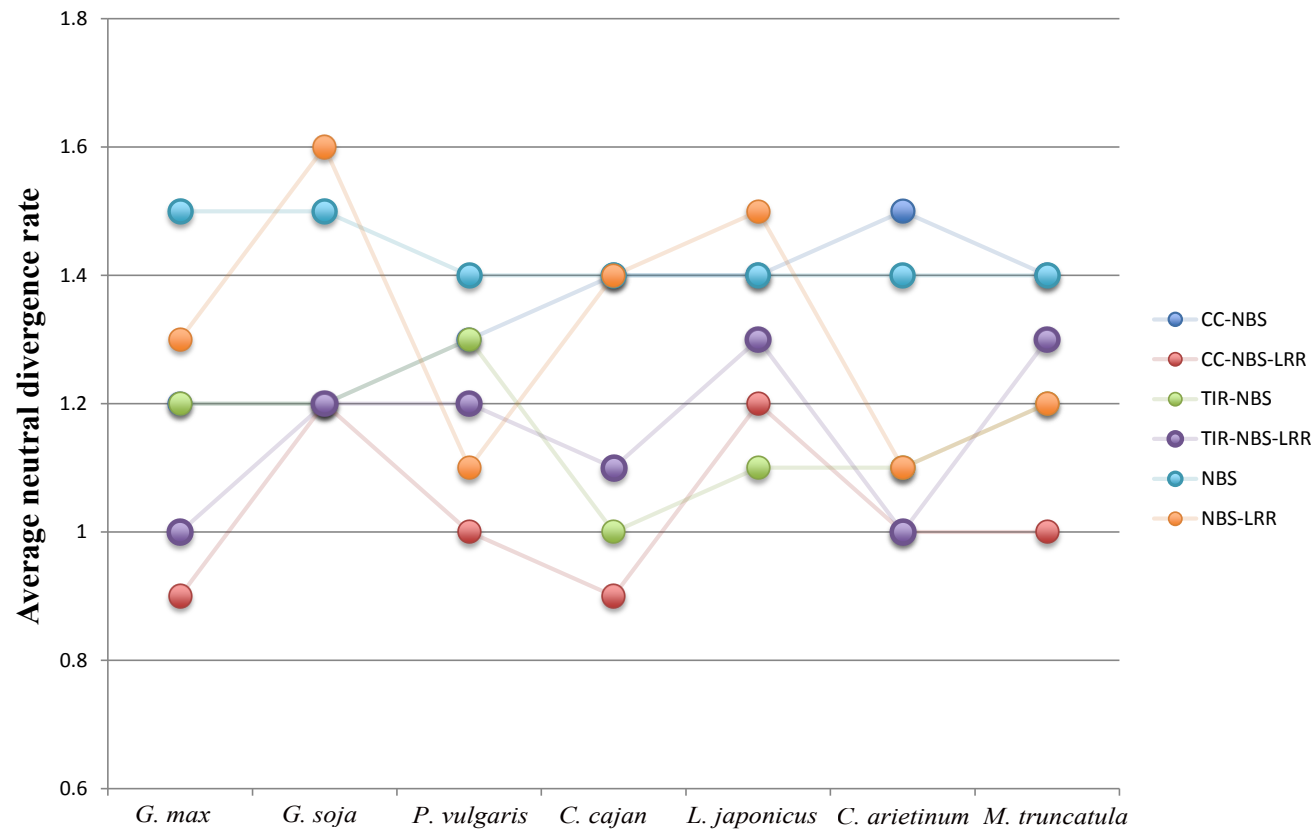

Supplement: Additional file 11: Figure S9. — The average neutral divergence rates of different domains in R-genes in the legume family. Note that some domains have identical values that there are some overlapping points in the figure. (PDF 93 kb) [file 12864_2016_2736_MOESM11_ESM.pdf]
